# The Application of Diffusion- and Perfusion-Weighted Magnetic Resonance Imaging in the Diagnosis and Therapy of Acute Cerebral Infarction

**DOI:** 10.1155/IJBI/2006/20164

**Published:** 2006-10-18

**Authors:** Ying Han, Enzhong Li, Jie Tian, Jian Chen, Huifang Wang, Jianping Dai

**Affiliations:** ^1^ Institute of Cognitive Neuroscience and Learning, Beijing Normal University, Beijing 100875, China; ^2^ Associated Laboratory, Beijing Tiantan Hospital, Beijing 100050, China; ^3^ Medical Image Processing Group, Institute of Automation, Chinese Academy of Sciences, P.O. Box 2728, Beijing 100080, China; ^4^ Department of Neurology, Shougang Hospital, Beijing University, Beijing 100041, China

## Abstract

Diffusion- and perfusion-weighted magnetic resonance imaging (DWI and PWI) was applied for stroke diagnose in 120 acute (< 48 h) ischemic stroke patients. At hyperacute (< 6 h) stage, it is difficult to find out the infarction zone in conventional T1 or T2 image, but it is easy in DWI, apparent diffusion coefficient (ADC) map; when at 3–6-hour stage it is also easy in PWI, cerebral blood flow (CBF) map, cerebral blood volume (CBV) map, and mean transit time (MTT) map; at acute (6–48 h) stage, DWI or PWI is more sensitive than conventional T1 or T2 image too. Combining DWI with ADC, acute and chronic infarction can be distinguished. Besides, penumbra which should be developed in meaning was used as an indication or to evaluate the therapeutic efficacy. There were two cases (< 1.5 h) that broke the model of penumbra because abnormity was found in DWI but not that in PWI, finally they recovered without any sequela.


Hindawi Publishing Corporation
International Journal of Biomedical Imaging
Volume 2006, Article ID 20164, Pages 1–11
DOI 10.1155/IJBI/2006/20164

### The Application of Diffusion- and Perfusion-Weighted Magnetic Resonance Imaging in the Diagnosis and Therapy of Acute Cerebral Infarction

**Ying Han,[1] Enzhong Li,[2, 3] Jie Tian,[3] Jian Chen,[3] Huifang Wang,[4] and Jianping Dai[2]**

[1] *Institute of Cognitive Neuroscience and Learning, Beijing Normal University, Beijing 100875, China*
[2] *Associated Laboratory, Beijing Tiantan Hospital, Beijing 100050, China*
[3] *Medical Image Processing Group, Institute of Automation, Chinese Academy of Sciences, P.O. Box 2728, Beijing 100080, China*
[4] *Department of Neurology, Shougang Hospital, Beijing University, Beijing 100041, China*

Received 4 January 2006; Revised 2 July 2006; Accepted 18 July 2006

Recommended for Publication by Lizhi Sun

Diffusion- and perfusion-weighted magnetic resonance imaging (DWI and PWI) was applied for stroke diagnose in 120 acute (< 48 h) ischemic stroke patients. At hyperacute (< 6 h) stage, it is difficult to find out the infarction zone in conventional T1 or T2 image, but it is easy in DWI, apparent diffusion coefficient (ADC) map; when at 3–6-hour stage it is also easy in PWI, cerebral blood flow (CBF) map, cerebral blood volume (CBV) map, and mean transit time (MTT) map; at acute (6–48 h) stage, DWI or PWI is more sensitive than conventional T1 or T2 image too. Combining DWI with ADC, acute and chronic infarction can be distinguished. Besides, penumbra which should be developed in meaning was used as an indication or to evaluate the therapeutic efficacy. There were two cases (< 1.5 h) that broke the model of penumbra because abnormity was found in DWI but not that in PWI, finally they recovered without any sequela.

Copyright © 2006 Ying Han et al. This is an open access article distributed under the Creative Commons Attribution License, which permits unrestricted use, distribution, and reproduction in any medium, provided the original work is properly cited.

### 1. INTRODUCTION

Stroke is a life-threatening disease complicated with long-term disability. About 85% of stroke is caused by cerebral ischemic infarction. An accurate diagnosis in acute stage is extremely important for its treatment and prognosis. DWI and PWI with their postprocessing technique can diagnose the brain infarction in very early stage.

DWI can identify ischemic core in acute stroke patients, abnormalities observed on DWI allow an early identification of severely ischemic brain regions that typically evolve into infarction and are thought to represent the irreversible infarct core. PWI [1, 2] using dynamic susceptibility contrast and its derivation parameters such as CBF, CBV, and MTT [3] enable the identification and characterization of perfusion deficits both within and importantly in regions surrounding the ischemic core; perturbations in perfusion can be analyzed from maps of CBF, CBV, and MTT through an ischemic region. Generally, PWI impaired areas are larger than the DWI lesions during the first hours of stroke evolution so the mismatch is computed by PWI minus DWI. The mismatch region between the abnormalities delineated on the initial diffusion and perfusion maps acquired in the acute stage of stroke have previously been reported to represent the ischemic penumbra; it is a reversible region that is still viable but will eventually evolve to infarction. Most pharmacological interventions in the acute phase of ischemic stroke are based on this region. The recently demonstrated reversibility of the diffusion lesion with thrombolytic therapy has enabled redefinition of the ischemic penumbra to include both the DWI/PWI mismatch and regions of the initial diffusion abnormality.

Some researchers [4] indicate that DWI/PWI mismatch may constitute not only the area at risk for infarction but also oligemic tissue with blood flow above the critical viability threshold that is not at risk. Therefore, it is crucial to quantify the importance of the perfusion deficit within the DWI/PWI mismatch area to distinguish the real area at risk from the oligemic tissue that does not constitute a target for thrombolysis, so quantitative measures of CBF and CBV may be useful for this purpose. In the infarct core predicting aspect, even though the diffusion lesion normally expands into the surrounding hypoperfused penumbra, a model of infarct evolution based on this simple mismatch parameter alone does not accurately predict the final infarct volume; some authors [5] have researched the new penumbra model to predict the final infarct core. In our study, it is validated that combining DWI and PWI, acute stoke can be diagnosed in 3–6 hours. Besides, penumbra which should be developed was used as an indication or to evaluate the therapeutic efficacy.

In this study, we found some cases in which the abnormity in DWI was found less than 1.5 hour of ictus but not found in PWI, and finally recovered without any sequela; this means that DWI lesions could be cured totally at the beginning of hyperacute stage. Besides, in the other stage of hyperacute infarction, the abnormity in DWI can be partially cured but still left an infarct core.

### 2. MATERIALS AND METHODS

### 2.1. Subjects one hundred and twenty acute stroke patients were selected from 350 stroke patients during March 1999 to March 2003 with the average age of 64-year old (28–80 years). Eighty six out of 120 patients suffered from brain infarction and 34 patients had transient ischemic attack (TIA).

### 2.2. MR scanning

One and half T superconductive magnetic resonance imaging scanner and a quadrature head coil were used (GE Medical System, Milwaukee, USA or Siemens, Erlangen, Germany). Sagittal, coronal, and axial sections of images were obtained with spin echo (SE) T1-weighted imaging pulse sequence (TR 440 ms, TE 11 ms, FOV 24 or 22 cm, matrix 256 × 256, slice thickness 6 or 5 mm, slice space 3 or 2.5 mm). The axial T2-weighted images were obtained with fast spin echo (FSE) pulse sequences (TR 4000 ms, TE 110 ms, FOV 24 or 22 cm, matrix 256 × 256, slice thickness 6 or 5 mm, slice space 3 or 2.5 mm); fluid attenuated inversion recovery (FLAIR) were obtained with FLAIR pulse sequence (TR 10000 ms, TE 126 ms, TI 2200 ms, FOV 24 or 22 cm, matrix 256 × 256, slice thickness 6 or 5 mm, slice space 3 or 2.5 mm), magnetic resonance angiography (MRA) images with 3 dimension time of flight (3D-TOF) method, and the diffusion weighted imaging was performed by using a single shot echo planar imaging (EPI) pulse sequence with $b$-value 1000 s/mm$^2$ (TR 2000 ms, TE 126 ms, TI 200 ms, flip angle 90, matrix 128 × 128, the other parameters were same as the T1-weighted images); perfusion-weighted images were acquired by using dynamic first pass bolus tracking of gadolinium diethyllenetriamine penta acetic acid (Gd-DTPA). An echo planar imaging (EPI) pulse sequence was used (TR 2000 ms, TE 60 ms, flip angle 90, matrix 128 × 128, slice thickness 6 or 5 mm, slice space 3 or 2.5 mm), Gd-DTPA bolus (0.2 mmol/kg) was administered by a high-pressure syringe specially for magnetic resonance imaging (MRI).

### 2.3. Data analysis

#### 2.3.1. Apparent diffusion coefficient (ADC) and exponential apparent diffusion coefficient (EADC)

ADC values were acquired according to Stejskal-Tanner equation [6]:

$$S_b = S_0 \cdot e^{-b \cdot D}, \qquad D = -\frac{1}{b} \ln \frac{S_b}{S_0}, \tag{1}$$

where $D$ is diffusion coefficient, $S_0$ is signal intensity of the images without $b$ factor, and $S_b$ is signal intensity of the images recorded by using various $b$ factors, $b$ is diffusion weighting factor given by

$$b = \gamma^2 G^2 \delta^2 (\Delta - \delta/3), \tag{2}$$

where $\gamma$ is gyromagnetic radio, $\delta$ is duration of gradient irradiation, and $\Delta$ is time between the rising edges of two diffusion sensitizing gradients.

Because the brain is anisotropic, diffusion restrictions are orientation dependent. The diffusion coefficient $D$, therefore, has $D_{xx}$, $D_{yy}$ and $D_{zz}$:

$$D = \frac{D_{xx} + D_{yy} + D_{zz}}{3}. \tag{3}$$

In human brain, we often use ADC instead of $D$ then (3) can be written as

$$\text{ADC} = \frac{D_{xx} + D_{yy} + D_{zz}}{3}. \tag{4}$$

The EADC can be calculated by equation

$$\text{EADC} = e^{-b \cdot \text{ADC}}. \tag{5}$$

Then, ADC or EADC maps can be generated according to the values of ADC or EADC.

Both lesion area and contralateral mirror area in the contralateral hemisphere were segmented in order to make a comparative and quantitative analysis. Segmentation was done on DWI and an average ADC value in the area was calculated.

### 2.4. Cerebral blood flow (CBF), cerebral blood volume (CBV), and mean transit time (MTT)

For the perfusion weighted imaging, at first, MR signal intensity must be converted to Gd-DTPA concentration as the following equation [7]:

$$C_m(t) = -K \cdot \ln \frac{S(t)}{S_0}, \tag{6}$$

where $C_m(t)$ is the measured concentration of Gd-DTPA with time; $K$ is a proportionality constant that is inversely proportional to the time of echo (TE) and depends on MR

scanner; $S(t)$ is MRI signal intensity with time; and $S_0$ is baseline of MR signal before injection of Gd-DTPA and after a steady-state magnetization has been achieved. $K$ will be countervailed if we use gradient echo planar imaging (EPI) pulse sequence and the same TE in acquiring all slices. It is considered that each tissue type such as large and small vessels will have the same proportionality constant when using gradient EPI pulse sequence.

When ideal instantaneous arterial injection of Gd-DTPA is taken in a tissue region, the following relation exists:

$$\frac{\text{CBV}}{\text{CBF}} = \frac{\int C(t)dt}{C_{\max}}, \quad (7)$$

where $C(t)$ is the concentration of Gd-DTPA in a tissue region and $C_{\max}$ is the maximum of this curve. $C(t)$ can be calculated as follows:

$$C(t) = C_m(t) \otimes^{-1} \text{AIF}(t), \quad (8)$$

where $C_m(t)$ is the measured tissue curve, AIF$(t)$ is the measured arterial input function (AIF) curve, $\otimes^{-1}$ represents deconvolution, thus CBV can be calculated as

$$\text{CBV} = \frac{\kappa}{\rho} \cdot \frac{\int C_m(t)dt}{\int \text{AIF}(t)dt}, \quad (9)$$

where $\kappa = (1 - \text{HCTLV})/(1 - \text{HCTSV})$, while, hematocrit in large vessels, HCTLV $= 0.45$; hematocrit in small vessels, HCTSV $= 0.25$; the density of brain tissues, $\rho = 1.04$ g/ml. If CBV is known,

$$\text{CBF} = \frac{\int C_m(t)dt}{\text{CBV} \cdot C_{\max}} \quad (10)$$

and then,

$$\text{MTT} = \frac{\text{CBV}}{\text{CBF}}. \quad (11)$$

### 2.5. *Arterial input function (AIF) and gamma variate fitting*

Gamma variate fitting was applied to denoise the data for tracer recirculation [8, 9]. AIF$(t)$ and tissue $C_m(t)$ curves were both fit to a gamma variate function using the Levenberg-Marquardt method [10],

$$\text{AIF}_{\text{fit}}(t) = -K(\chi - \Delta)^{\alpha} \cdot e^{-(\chi-\Delta)/B} \cdot F_{\text{step}}(\chi - \Delta) \quad (12)$$

or

$$C_{\text{fit}}(t) = -K(\chi - \Delta)^{\alpha} \cdot e^{-(\chi-\Delta)/B} \cdot F_{\text{step}}(\chi - \Delta), \quad (13)$$

where AIF$_{\text{fit}}(t)$ and $C_{\text{fit}}(t)$ are fitted AIF$(t)$ and $C_m(t)$ curves, $K$ is a constant, $\chi$ is image number, $\Delta$ is the delay between image 0 and the arrival of the bolus, $\alpha$ and $B$ are gamma variate parameters, and $F_{\text{step}}$ is step function defined by

$$F_{\text{step}} = \begin{cases} 1(\chi - \Delta) \geq 0, \\ 0(\chi - \Delta) < 0. \end{cases} \quad (14)$$

All the time points less than 30% of the maximum after the peak of the AIF$(t)$ and $C_m(t)$ curves were not used in the fit. In the infarct areas, the threshold 30% was too low and was increased, until the $\chi 2$ value of gamma variate fit was within ±10% of the value in from healthy mirror area.

### 2.6. *Segmentation*

The values were calculated according to (6) to (14), and then the quantitative maps of CBV, CBF, and MTT were obtained. The segmentation was made in the lesion areas by DWI, ADC, CBV, CBF, and MTT. Then we registered and compared those segmented maps; the difference of the areas between the images (DWI, ADC to CBV, CBF, or MTT) was penumbra.

In comparison, an automatic segmentation method was used in both lesion and its contralateral mirror area from the images of stroke patients as proposed by Rajapakse [11]. The segmentation was a multistage process, involving image preprocessing, global and local registrations between the anatomical brain atlas and the patient's MRI, and finally segmentation of infarction based on region splitting and merging as well as multiscale adaptive statistical classification.

At first, the images had been preprocessed, including noise removing by nonlinear anisotropic diffusion filter, detection of the site and size of infarction volume by using spatial information, intensity gradient and contextual information of brain atlas, and making registration; therefore the brain atlas corresponded to the patient image by both global and local registrations.

After preprocessing, a multiscale adaptive statistical classification model was applied to segment the infarction lesion and normal contralateral mirror area. The multiscale approaches in image analysis were used for describing images at different levels of resolution [12, 13]. To overcome the drawback of blurring important image features, nonlinear scale space was employed according to Perona and Malik's partial differential equation model [14],

$$\frac{\partial I_l}{\partial t} = \text{div}\left(c(i,t)\nabla I_l\right) = c(i,t)\Delta I_l + \nabla c \cdot \nabla I_l, \quad (15)$$

where $I_l$ stands for luminance of the images which depend on position $i$ and $t$ scale level, $c(i,t)$ is diffusion coefficient, which is assumed as variant dependent of the space location, div is divergence operator, $\nabla$ is gradient operator, $\Delta$ is Laplacian operator.

The diffusion coefficient was chosen locally as a function of the magnitude for the gradient of the brightness,

$$c(i,t) = g\left(\left|\nabla I_l(i,t)\right|\right). \quad (16)$$

Different functions were used for $g(\cdot)$ giving perceptually similar results. The images in this paper were obtained by using

$$g(\nabla I_l) = e^{(-(|\nabla I_l|/Ks)^2)}. \quad (17)$$

The constant $Ks$ was fixed by hand at some fixed value or by the "noise estimator" [15].

The different scale level $t$ represents the different image spatial resolution. Scale level $t = 0$ indicates the original image. With the increasing scale level, the image was more blurring with less information. It is reasonable to think that local and high-resolution scale information is related to general and low-resolution scale information. This enables us to extract image structure.

Segmentation was started by using multiscale statistical classification (MSSC). Using (15), (16), (17), and anisotropic diffusion filter, a series of images was obtained with different resolutions. The nonlinear anisotropic diffusion effectively countered RF inhomogeneity by smoothing the brain regions. The level of detail decreased while the scale level increased. The blurred image series is represented by $y(t)$, $t \in \mathbb{N}$, $\mathbb{N} = \{1, 2 \ldots n\}$ ($t$ is the number of scale level, and $n$ is the maximum of $t$). If the segmentation $x(t+1)$ was acquired at scale level $t+1$, the parameters $\theta(t+1)$ can be estimated accordingly. With known parameters $\theta(t+1)$ at scale level $t$, the new segmentation of image $y(t)$ was obtained at scale level $t$. After certain iterations of segmentation and parameter estimation, by reaching the original image ($t = 0$) with the finest spatial resolution of the image series, the final optimal segmentation was achieved. The segmentation can be expressed in a two-step process:

$$x(t) = \arg\min_{x} U\big(x(t+1) \mid y(t), \theta(t+1)\big); \tag{18}$$

here, the most likely condition of segmentation was estimated and the model parameters for each class were given:

$$\theta(t) = \arg\max_{\theta} P\big(y(t) \mid x(t), \theta(t+1)\big); \tag{19}$$

here, the model parameters were estimated and the segmentation was given.

The measurement model parameters were chosen to maximize the likelihood of the image data. The MSSC procedure converged to a minimum of energy function $U(t)$ as shown in (20) after tissue classes were determined over the image for all iterations,

$$\begin{aligned} U(x) = {} & \frac{1}{2} \sum_{k} \sum_{i \in R_k} \left( \frac{y_i - \mu_{k,i}}{\sigma_{k,i}} \right)^2 \\ & + \sum_{k} \sum_{i \in R_k} \log\left(\sigma_{k,i}\right) + \beta \sum_{c \in C} V_c(x), \end{aligned} \tag{20}$$

$Ui(t)$ denotes the local energy, which is at pixel site $i$ at scale level $t$, and it is given by

$$\begin{aligned} U_i(t) = \arg\min_{k} \Bigg\{ & \frac{1}{2} \left( \frac{y_i(t) - \mu_{k,i}(T+1)}{\sigma_{k,i}(t+1)} \right)^2 \\ & + \log\left(\sigma_{k,i}(t+1)\right) + \beta V_c\left(x_i(t+1)\right) \Bigg\}, \end{aligned} \tag{21}$$

where $\mu_{k,i}(t)$ and $\sigma_{k,i}(t)$ are $\mu_{k,i}$ and $\sigma_{k,i}$ of the image at scale level $t$, and $V_c(x_i(t))$ is the number of pixels where $x_j(t) = x_i(t)$ for $i$ and $j$ are in the clique $C$. Convergence was decided when the number of changes of the tissue classes at the pixel sites droped below a certain threshold.

In DWI images, infarction lesion showed up with the increased signal while the intensity of nerve tracts was also high. It is difficult to accurately detect the site and size of infarction volume in stroke patients and also hard to identify nerve tracts from infarction lesion due to the overlapping intensities. We resolved the problem by comparing the DWI image with the anatomical digital atlas. After classifying a pixel by (21), we modified the result by an adjusting factor $\alpha(t) = \{\alpha_i(t),\ i \in I\}$. When a pixel at site $i$ was classified as infarction lesion, we calculated the adjusting factor $\alpha_i(t)$ at site $i$,

$$\alpha_i(t) = \left| y_i(t) - r_i(t) \right|, \tag{22}$$

where $r_i(t)$ is the intensity of the digital atlas at site $i$ of scale level $t$, and $\alpha_i(t)$ is the absolute value of the difference between $y_i(t)$ and $r_i(t)$. Here we assumed $T$ is the threshold for $\alpha_i(t)$, and we set the value of $T$ by experience. If $\alpha_i(t) < T$, we suppose that the classification is confused and the pixel is not in the region of infarction lesion.

From (19), we got the measurement model parameter set $\theta(t) = \{\theta i(t),\ i \in I\}$ at scale level $t$, where $\theta_i(t) = \{\theta k,\ i(t) = (\mu_{k,i}(t), \sigma_{k,i}(t)),\ k \in \Lambda\}$, by differentiating $\log p(y(t) \mid x(t), \theta(t+1))$ with respect to $\mu_{k,i}(t)$ or $\sigma_{k,i}(t)$ and made it equate to zero. The estimations $\mu_{k,i}(t)$ and $\sigma_{k,i}(t)$ are given by

$$\begin{aligned} \mu_{k,i}(t) &= \mu_k(t) = \frac{\sum_{i \in R_k} y_i(t)}{\left| R_k(t) \right|}, \\ \sigma^2_{k,i}(t) &= \sigma^2_k(t) = \frac{\sum_{i \in R_k} \left(y_i(t) - \mu_{k,i}(t)\right)^2}{\left| R_k(t) \right|}, \end{aligned} \tag{23}$$

where $R_k(t)$ denotes the region or the set of all pixel sites belonging to tissue class $k$ at scale level $t$.

At last, in order to estimate the image segmentation with highest spatial resolution (at lowest scale level, $t = 0$), a series of consecutive segmentations at different scale levels were needed. A starting configuration of $x(n)$ was needed by a rough segmentation, which was initially obtained by region splitting and merging at the highest scale level $n$. Before region splitting and merging, we confirmed the number of classes $K$ and the maximum number of total regions. We splited the blurred image into M/16 regions, where M was the total number of pixels in the image; and then we merged the regions into $K$ tissue classes. Based on the initial rough segmentation, we can get accurate segmentation of the original image with the highest spatial resolution in scale space by MSSC method with (18), (19), (21), (22), and (23).

### 3. RESULTS

One hundred and twenty patients were divided into 3 groups according to time from stroke ictus to receiving treatment. The first group was diagnosed within 3 hours, in which almost all patients can be treated with thrombolytic therapy; the second group was diagnosed between 3 to 6 hours, in which some of the patients received thrombolytic therapy; and the third 6 to 48 hours, only a few had thrombolytic therapy (see Table 1).

Table 1: Patient groups according to time from stroke onset to being treated.

| Patients group | Group 1 | | Group 2 | Group 3 |
|---|---|---|---|---|
| | < 1.5 h | 1.5–3 h | 3–6 h | 6–48 h |
| Brain infarction | 2 | 8 | 11 | 65 |
| Transient ischemic attack | 3 | 7 | 10 | 14 |

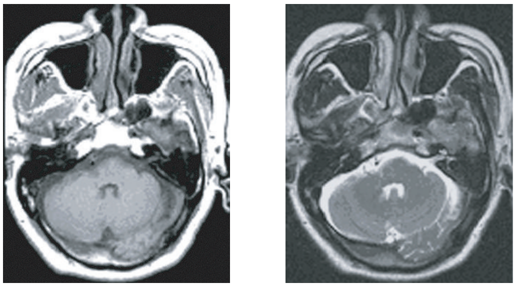

(a) (b)

Figure 1: A 46-year-old male. Hyperacute stage (1.5 hour). T1-weighted image (a) and T2-weighted image (b). No abnormality was found.

### 3.1. The signal intensity

In conventional T2- or T1-weighted images, the cerebral infarction showed normal signal in both the first and second groups (Figures 1, 4). Hyperintense in T2-weighted images or FLAIR images were observed in the third group. In this study, abnormal signals were not found within 14 hours after stroke onset. It showed that the infarction region signals were hyperintense obscurely.

In DWI, the signals showed hyperintense in all three groups. ADC values were declined obviously in hyperacute (< 6 h) and acute (6–48 h) stage of stroke, and signals of lesion areas were hypointense in ADC maps (Figures 2, 4, 5). After that, however, ADC gradually recovered with the development of the disease, and changed to hyperintense in ADC maps.

In PWI, there were hyperintense signals in MTT map (Figure 5) and hypointense in both CBV and CBF maps generally. In a few cases, there was normal signal intensity or hyperintensity in MTT map.

With MRA, a signal loss in the left cerebral middle artery was found (Figure 5).

### 3.2. The sensitivity of DWI, PWI, and T2 WI

Among infarction patients, in the first group, the cerebral infarction areas' signals changed to hyperintensity only in diffusion- or perfusion-weighted images (Figure 2), they were normal in conventional images such as T2- or T1-weighted images (Figure 1). ADC declined obviously, and there was a remarkable hypointensity in ADC map (Figure 2). But in this group, there was one case failed for DWI detection. Therefore, the sensitivity of DWI or PWI was 75% in this group.

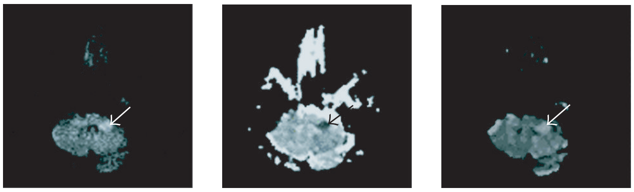

(a) (b) (c)

Figure 2: The same patient as Figure 1. Hyperacute stage (1.5 hour). The images are DWI (a), ADC map (b), and EADC map (c), respectively. The hyperintense signals in DWI, EADC map, and hypointense signal in ADC map can be seen.

In the second group, there were hyperintense signals in diffusion- or perfusion-weighted images (Figure 5); no abnormal signals were found in conventional T2- or T1-weighted images. ADC declined obviously, and there was a remarkable hypointensity in ADC map (Figure 5). In this group, the sensitivity of DWI or PWI went to 100%.

In the third group, the infarction areas of all patients were clearly detected with the diffusion-weighted imaging and ADC mapping techniques. Besides, with conventional T2-weighted images and FLAIR images, only 36 cases were detected (Figure 4). The sensitivity of DWI or PWI was 100% and T2 WI was 76%.

### 3.3. DWI-PWI mismatch

DWI-PWI mismatch was also noticed (Figure 5). There was the DWI-PWI mismatch called penumbra around the infarction area (Figure 5). For instance, if the hyperintense areas in MTT map were larger than the ones in DWI, the larger part was penumbra and could be cured by an appropriate thrombolytic therapy.

### 3.4. The continuous observation of the lesion after the therapy

We made a continuous observation after thrombolytic therapy, and found that DWI-PWI mismatch was changed dynamically. In the subgroup 1.5 hour of group 1, there was no DWI-PWI mismatch, all of the abnormal areas disappeared gradually after treatment; in the subgroup < 3 h, we found the DWI-PWI mismatch, and almost all of the abnormal areas were changed to normal after the treatment; some parts of the abnormal areas recovered in group 2, and only a few areas in group 3. In this study, we also calculated that the volume of abnormal areas before and after the therapy by segmentation and found that the lesion disappeared in the subgroup 1.5 hour and more than 90% of the lesion areas recovered in the subgroup < 3 hours of group 1. Within 6 hours, the recovered areas were about 70 to 80% and the areas dropped to about 40% at 8 hours (Figure 6).

### 3.5. The therapeutic efficacy in the different groups

We selected and analyzed the three-group data within the first 12 hours of infarction, compared the lesion areas before

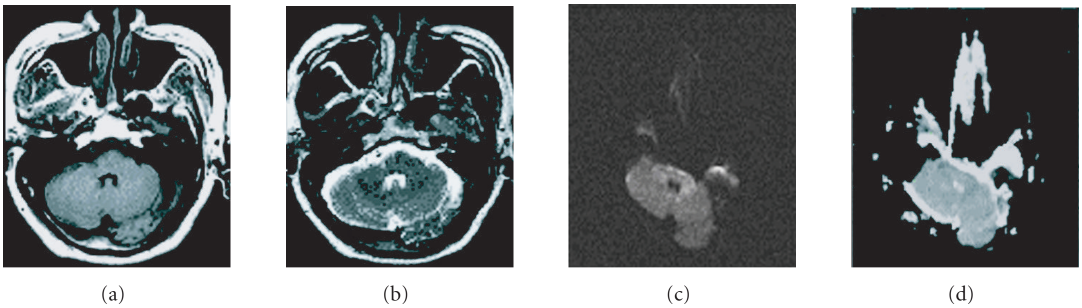

Figure 3: The same patient as in Figures 1 and 2. Hyperacute stage (7 days). T2-weighted image (a), T1-weighted image (b), DWI (c), and ADC map (d). No abnormity was found in each figure. The abnormity in DWI and ADC map have disappeared.

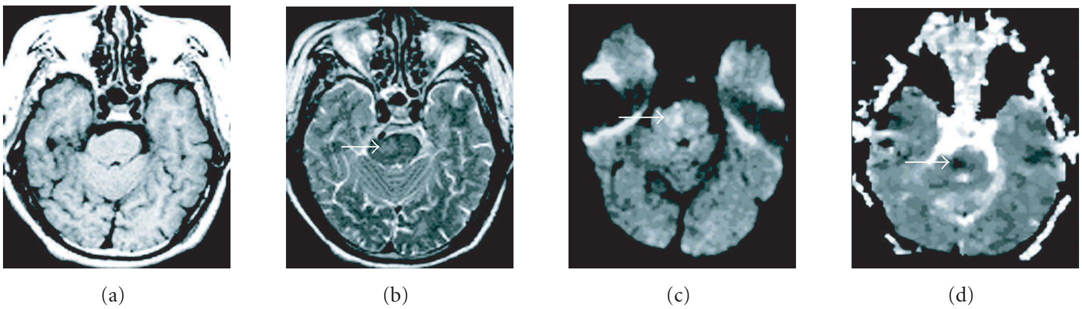

Figure 4: A 68-year-old male, brainstem infarction in acute stage (14 hours). The images are T1-weighted image (a), T2-weighted image (b), DWI (c), and ADC map (d), respectively. No clear abnormal signal can be seen in T1-weighted image, but slightly hyperintense signal in T2-weighted image, hyperintense signal in DWI, and hypointense signal in ADC map.

and after the therapy, and found that the thrombolytic therapy was very effective in the first 3 hours, that is, group 1, most of the patients can be recovered. In group 2, which is 3–6 hours after infarction, there was still infarction core left and the patients have different sequelae after the therapy. Besides, the risk of hemorrhage was increased. In group 3, the thrombolytic therapy was not effective, the percentage of the recovery after the therapy was less than 27% (Table 2, Figure 7).

### 4. DISCUSSION

### 4.1. *The value of DWI and PWI in the diagnosis of hyperacute stroke*

DWI and PWI bring great promise of increasing the sensitivity of diagnosing infarction; and are considered as a powerful predictor of stroke [16]. With DWI technology, the constant motion of water molecule in the brain, the diffusion of water molecule within extracellular space, and between intracellular and extracellular space can be measured [17]. When diffusion is restricted, DWI signal shows hyperintensity, as occurs in cytotoxic damage from ischemia, inflammation, trauma, or tumor [18]. DWI is still valuable from spin density and relaxation times T1 and T2, which we know from the equation above; therefore, the hyperintense lesion on DWI may reflect a strong T2 effect instead of reduced water diffusion. In order to diminish T2 effect, the diffusion coefficient $D$ was calculated as the macroscopic mean of heterogeneous tissue compartments with varying diffusion properties and it was referred to as the apparent diffusion coefficient (ADC). ADC plays an important role in stroke diagnosis, especially at acute stage. Because of diminished T2 effect, some small hyperintense lesion can be identified; even they have a bright background such as those near the subarachnoid space. ADC values may also provide useful information in predicting perfusion status [19]. Some authors studied infarction from 5 hours after onset of clinical symptoms to 12 months and then made a time course of ADC. They found that at first ADC declined, approximately 28 hours to a minimum, then ADC reincreased and reached a “pseudonormalization” after approximately 5 days; ADC values went much higher in chronic infarctions. Neither localization nor size of infarctions showed a significant influence on this time course [20].

Combining DWI with ADC, we can make an accurate diagnosis of stroke, especially in the hyperacute ($< 6$ h) stage. In our study, we found the earliest case just in 1.5 hour after onset of clinical symptoms by DWI and ADC map. This confirms that DWI and ADC can detect the infarct lesion in hyperacute stage; even sometimes DWI fails to identify the infarction at one case we have experienced. Pedraza et al. reported that a 70-year old woman had a Wallenberg’s syndrome and a medulla lateral infarction. However DWI

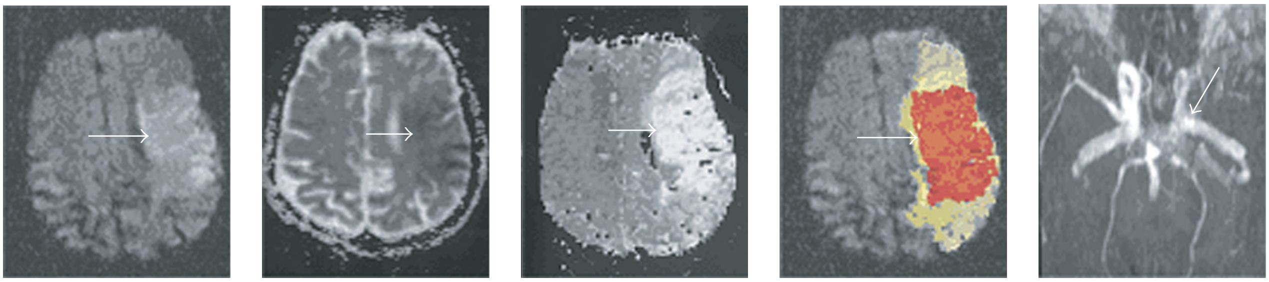

Figure 5: A 55-year-old female, brainstem infarction in hyperacute stage (6 hours). The images are DWI (a), ADC map (b), MTT map (c), penumbra map (d) and MRA (e), respectively. The abnormal signal was located in left frontal lobe, which showed hyperintense signal in DWI, and hypointense signal in ADC map. In MTT map, the hyperintense signal was also seen and the abnormal region seemed larger than that in DWI. The mismatch of DWI and PWI was then processed and the penumbra map was obtained (the red part implied the infarction part and yellow one meant penumbra). MRA showed that there was a signal loss in the left cerebral middle artery.

Table 2: The penumbra change and thrombolytic therapy.

| Patients group | Group 1 | | Group 2 | Group 3 | |
|---|---|---|---|---|---|
| | < 1.5 h | 1.5–3 h | 3–6 h | 6–9 h | 9–12 h |
| Recovery percentage for subgroup | 100% | 97% | 73% | 41% | 13% |
| Recovery percentage for group | 98% | | 73% | 21% | |
| After thrombolytic therapy | Total or most of lesion recovered | | Partial recovered but had a risk | Thrombolytic therapy is not recommend | |

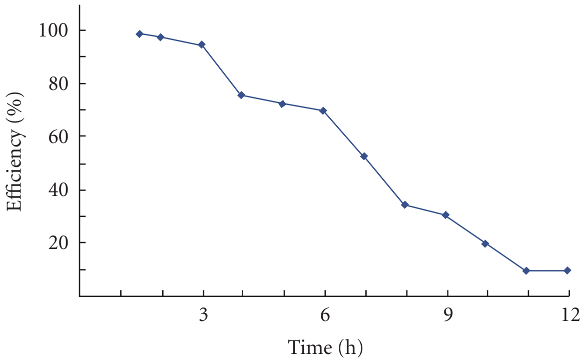

Figure 6: Relationship between therapeutic efficacy and the onset time. The result shows that the earlier the treatment is taken, the better the therapeutic efficacy is. The patient can totally recover if he was treated within 1.5 hour from stroke onset. As time goes by, the patient has less chance to recover.

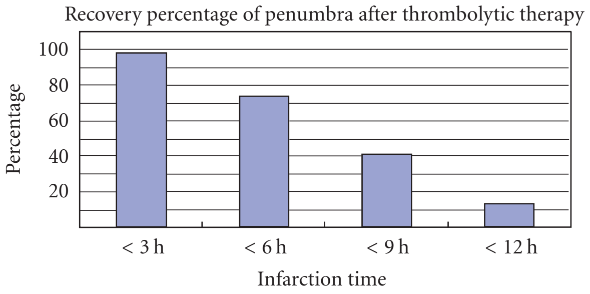

Figure 7: The therapeutic efficacy of the three groups. The data were selected only the first 12 hours of infarction. The result shows that the earlier the treatment is taken, the better the therapeutic efficacy is. If the thrombolytic therapy was given within 3 hours, it is very effective and the most of the patients can be recovered.

showed normal signal 10 hours after onset of symptoms [21]. The sensitivity of DWI is reduced during the first 24 hours. False negative diagnoses mainly happen in small infarctions of posterior territory. DWI is a powerful diagnostic means of stroke, even it cannot detect all the early infarction. Some authors reported that it had 85.7% in sensitivity and 95.7% in specificity [16]; the sensitivity of DWI in our study was 87.5% within the first 6 hours. The sensitivity of DWI is not only lower in the early stage [22], but also associated with the infarct territories. In the posterior territory, there are small infarctions; therefore, false negative diagnosis happens during the first 24 hours [21]. According to our work, the small lesion mostly located in the brainstem. We have a case that the very small lesion was not found in DWI by 3 hours after infarction.

Combining DWI with ADC, acute and chronic infarction can be distinguished. The signal is hyperintense because of restricted diffusion and ADC value decline in acute stage of infarction; the signal will change to hypointense as time goes by because the tissue will be destroyed and cannot restrict diffusion any more; ADC value goes up. T2-weighted images and FLAIR images cannot diagnose the infarction in hyperacute (< 6 h) stage. Lesions were found at more than 14 hours after onset of symptoms in T2-weighted images or FLAIR images in our study. Other reports may be a little bit earlier, for example, 8 or 10 hours. These images are not useful to thrombolytic therapy, because the thrombolytic therapeutic work generally should be done within 3 hours after onset of clinical symptoms [23].

It is difficult to distinguish blood pool in MRI, because the cerebral intravascular space is only 5% of the brain. PWI technology, which is based on the differential velocities of water molecule, can amplify the MR signals by using the contrast media [24], so it can delineate abnormal tissue perfusion. Among fast MR imaging techniques, EPI achieves the best temporal resolution to monitor cerebral vascular transit of magnetic susceptibility contrast media. Compared with the contralateral normal hemisphere, the hemodynamic consequence on perfused part of an injured hemisphere is a delayed and prolonged signal loss. Relative quantification of CBV may be obtained by integration of the area under the curve of signal evolution during the pass of the bolus [25].

Ideally, AIF curve should come up and then down and finally back to the baseline. One of the purposes of gamma variate fitting is to remove the influence of recirculation. Besides, some authors used deconvolution method and found that using this method maintained relatively high specificity not only in ischemic stroke group but also in the stenosis group [26].

The signals in both hyperacute and acute infarctions are hyperintense in MTT, hypointense in CBV and CBF. Because the supplying arteries are occluded in the stroke, the values of CBV and CBF go down, and MTT prolonged. Generally, the lesion areas in hyperacute stage of stroke measured in PWI are much larger than those in DWI, but the larger part areas will change gradually and finally to the same size as those of DWI. This mismatch region has been suggested to be "tissue at risk" or be called penumbra. The PWI-DWI mismatch suggests the presence of salvageable tissue in the brain.

### 4.2. The importance of early diagnosis to the effective therapy

DWI and PWI technologies play an important role in stroke treatment especially when stroke at hyperacute and acute stages.

An accurate early diagnosis can seize time and chance for stroke treatment. Two typical patients were diagnosed for stroke in 1.5 hour of onset symptoms and of course immediately followed by proper medication. The patients recovered without sequelae. The earlier the diagnosis is made, the better chance the patients have.

Penumbra can be used to evaluate the therapeutic efficacy. Penumbra is the abnormal brain tissue with dysfunction but without destruction. The tissue can be rescued by the treatment in time. Penumbra will become infarction if the lower blood reperfusion persists. The main purpose of the thrombolysis and other therapies is to protect penumbra from infarction. For this reason, we can examine the penumbra by comparing the areas before and after treatment to observe and evaluate therapeutic efficacy.

Since the beginning of 1990's, tissue plasminogen activator (tPA) has been used in stroke and it significantly improves neurological and functional outcome in stroke patients who were treated within 3 hours of symptom onset. The treatment window is expanded to 6 hours by PWI and DWI diagnoses, if the patients met one of the following: (1) the early PWI lesion estimates the region of acute dysfunctional brain tissue, whereas the acute DWI lesion corresponds to the core of the early infarction; (2) the mismatch between the acute PWI lesion and the smaller DWI lesion represents potentially salvageable brain tissue (an estimate of the ischemic penumbra); and (3) in patients with a PWI/DWI mismatch, early reperfusion is associated with substantial clinical improvement and reversal or reduction of DWI lesion growth [27, 28]. Besides, others considered that DWI-PWI mismatch ratio (PWI-DWI/PWI×100%) was correlated with the initial neurological severity, and that the rescued ratio (PWI-T2/PWI × 100) was an objective indicator of the efficacy of the treatment [24].

The result (Table 2) of our study shows, in acute stroke patients, accurate early diagnosis is important for treatment; otherwise, the pathological change is difficult to resume. Two patients receiving a medical examination in 1.5 hour after ictus have already recovered after treatment; we found that their pathological change has already totally disappeared by MRI examination (Figures 1–3).

Taking treatment by 3–6 hours after ictus, the pathological region obviously reduces, the abnormal signal intensity of about 70% legion areas became normal (Table 2, Figure 7).

Some patients treated by 6 hours after stroke onset, the ischemic penumbra have reduced, but the change is not very obvious because the most ischemic areas have already changed into infarct areas, and then destroyed brain tissues (Table 2, Figures 4, 7).

The results we educed are similar to the results obtained in animal's experiments [29].

### 4.3. The model of penumbra, the opportunity for therapy, and the therapeutic efficacy in three groups

Conventionally the penumbra was considered as the mismatch of DWI and PWI. But recently we know that the recovery can also occur in the abnormal area in DWI. The percentage depends upon the time of the treatment. For instance, in group 1 of our study, two typical ischemic stroke patients (< 1.5 h, subgroup 1), whose DWI showed hyperintense signals (abnormity) and PWI showed nearly normal signals, finally recovered without sequelae. These two cases broke the penumbra model. According to conventional penumbra model, the abnormity showed in DWI is the infarction zone and cannot be rescued, but in subgroup 1, the infarction zone has totally disappeared after the treatment. Therefore, the model of penumbra needs to be developed.

Besides, some patients in the second group, that is, at 3 to 6-hour stage, should be analyzed in detail according to the patient's condition. In our study, we found that the patient's lesion areas were different even in the same stage. For instance, there were some patients in group 2, at > 3-hour stage and were not treated with thrombolytic therapy according to the conventional model. After we analyzed their conditions, selected some of them whom we considered to be suitable for the therapy, and treated them, about 70 to 80% of their lesion areas recovered. From the results above, we consider that the time of infarct, the lesion situation in the brain, and the patient's whole condition should be analyzed synthetically. The infarct time is not an absolute index for the thrombolytic therapy.

It is noticeable for us to pay more attention to the hemorrhge risk during the treatment of thrombolysis, especially in group 2. In this group, the risk of hemorrhage is much more than that in group 1. For this reason, when a patient is treated at less than 3-hour stage, it is sensible for thrombolytic therapy, but if it is in the stage of more than 3 hours and the patient has the tendency of the hemorrhage, it is absolutely unadvisable (Table 2).

We recorded the time for treatment and compared the therapeutic efficacy of the acute stroke patients. The only criterion we used for judging the therapeutic efficacy is the infarct volume change before and after treatment. The results show that the earlier the treatment is taken, the better the therapeutic efficacy is. For instance, the patient can totally recover in subgroup 1 of group 1, that is, if he was treated within 1.5 hour after stroke ictus. As time goes by, the patient has less chance to recover (Table 2, Figures 6, 7).

### 4.4. Transient ischemic attack (TIA)

TIA is a transient ischemic disease and it is high risk for stroke. One research has reported that almost half of the patients with the clinical syndrome of TIA changed to infarction [30]. The risk may reduce by appropriately therapy. Quantitative DWI provides sensitivity compared with conventional DWI in diagnosing transient cerebral ischemia. Both conventional DWI and quantitative DWI were applied on TIA patients, and we found that ADC map in some areas declined slightly at the early start but returned normal signals after 48 hours. Further study will be conducted.

### ACKNOWLEDGMENTS

This paper is supported by the National Science Fund for Distinguished Young Scholars of China under Grant no. 60225008, the Special Project of National Grand Fundamental Research 973 Program of China under Grant no. 2002CCA03900, the National High Technology Development Program of China under Grant no. 2002AA234051, the National Natural Science Foundation of China under Grant nos. 60172057 and 30270403, the Science Fund for Young Scholars and Returned Visiting Scholars of Beijing Municipality, and the Special Project for Beijing Outstanding Talents across the Century.

### REFERENCES

[1] A. M. Smith, C. B. Grandin, T. Duprez, F. Mataigne, and G. Cosnard, "Whole brain quantitative CBF and CBV measurements using MRI bolus tracking: comparison of methodologies," *Magnetic Resonance in Medicine*, vol. 43, no. 4, pp. 559–564, 2000.

[2] A. M. Smith, C. B. Grandin, T. Duprez, F. Mataigne, and G. Cosnard, "Whole brain quantitative CBF, CBV, and MTT measurements using MRI bolus tracking: implementation and application to data acquired from hyperacute stroke patients," *Journal of Magnetic Resonance Imaging*, vol. 12, no. 3, pp. 400–410, 2000.

[3] C. B. Grandin, T. P. Duprez, A. M. Smith, et al., "Which MR-derived perfusion parameters are the best predictors of infarct growth in hyperacute stroke? Comparative study between relative and quantitative measurements," *Radiology*, vol. 223, no. 2, pp. 361–370, 2002.

[4] C. B. Grandin, T. P. Duprez, A. M. Smith, et al., "Usefulness of magnetic resonance-derived quantitative measurements of cerebral blood flow and volume in prediction of infarct growth in hyperacute stroke," *Stroke*, vol. 32, no. 5, pp. 1147–1153, 2001.

[5] S. E. Rose, J. B. Chalk, M. P. Griffin, et al., "MRI based diffusion and perfusion predictive model to estimate stroke evolution," *Magnetic Resonance Imaging*, vol. 19, no. 8, pp. 1043–1053, 2001.

[6] E. O. Stejskal and J. E. Tanner, "Spin diffusion measurements: spin echoes in the presence of a time-dependent field gradient," *The Journal of Chemical Physics*, vol. 42, no. 1, pp. 288–292, 1965.

[7] A. M. Smith, C. B. Grandin, T. Duprez, F. Mataigne, and G. Cosnard, "Whole brain quantitative CBF, CBV, and MTT measurements using MRI bolus tracking: implementation and application to data acquired from hyperacute stroke patients," *Journal of Magnetic Resonance Imaging*, vol. 12, no. 3, pp. 400–410, 2000.

[8] T. Hagen, K. Bartylla, and U. Piepgras, "Correlation of regional cerebral blood flow measured by stable xenon CT and perfusion MRI," *Journal of Computer Assisted Tomography*, vol. 23, no. 2, pp. 257–264, 1999.

[9] J. R. Petrella, C. DeCarli, M. Dagli, et al., "Assessment of whole-brain vasodilatory capacity with acetazolamide challenge at 1.5 T using dynamic contrast imaging with frequency-shifted burst," *American Journal of Neuroradiology*, vol. 18, no. 6, pp. 1153–1161, 1997.

[10] D. W. Marquart, "An algorithm for least squares estimation of nonlinear parameters," *Journal of the Society of Industrial and Applied Mathematics*, vol. 11, no. 2, pp. 431–441, 1963.

[11] J. C. Rajapakse, J. N. Giedd, and J. L. Rapoport, "Statistical approach to segmentation of single-channel cerebral MR images," *IEEE Transactions on Medical Imaging*, vol. 16, no. 2, pp. 176–186, 1997.

[12] J. A. Schnabel and S. R. Arridge, "Multiscale shape description of MR brain images using active contour models," in *Medical Imaging 1996: Image Processing*, vol. 2710 of *Proceedings of SPIE*, pp. 596–606, Newport Beach, Calif, USA, February 1996.

[13] O. D. Escoda, A. Petrovic, and P. Vandergheynst, "Segmentation of natural images using scale-space representations: a linear and a non-linear approach," in *Proceedings of 11th European Signal Processing Conference (EUSIPCO '02)*, vol. 3, pp. 481–484, Toulouse, France, September 2002.

[14] P. Perona and J. Malik, "Scale-space and edge detection using anisotropic diffusion," *IEEE Transactions on Pattern Analysis and Machine Intelligence*, vol. 12, no. 7, pp. 629–639, 1990.
[15] J. Canny, "Computational approach to edge detection," *IEEE Transactions on Pattern Analysis and Machine Intelligence*, vol. 8, no. 6, pp. 679–698, 1986.
[16] J. F. Arenillas, Á. Rovira, C. A. Molina, E. Grivé, J. Montaner, and J. Álvarez-Sabín, "Prediction of early neurological deterioration using diffusion- and perfusion-weighted imaging in hyperacute middle cerebral artery ischemic stroke," *Stroke*, vol. 33, no. 9, pp. 2197–2203, 2002.
[17] D. Le Bihan, E. Breton, D. Lallemand, M.-L. Aubin, J. Vignaud, and M. Laval-Jeantet, "Separation of diffusion and perfusion in intravoxel incoherent motion MR imaging," *Radiology*, vol. 168, no. 2, pp. 497–505, 1988.
[18] S. K. Mukherji, T. L. Chenevert, and M. Castillo, "Diffusion-weighted magnetic resonance imaging," *Journal of Neuro-Ophthalmology*, vol. 22, no. 2, pp. 118–122, 2002.
[19] W. Lin, J.-M. Lee, Y. Z. Lee, K. D. Vo, T. Pilgram, and C. Y. Hsu, "Temporal relationship between apparent diffusion coefficient and absolute measurements of cerebral blood flow in acute stroke patients," *Stroke*, vol. 34, no. 1, pp. 64–70, 2003.
[20] F. Ahlhelm, G. Schneider, M. Backens, W. Reith, and T. Hagen, "Time course of the apparent diffusion coefficient after cerebral infarction," *European Radiology*, vol. 12, no. 9, pp. 2322–2329, 2002.
[21] S. Pedraza, T. Osuna, A. Dávalos, J. Teruel, J. Vera-Sancho, and L. Inaraja, "False negative diffusion in acute ischemic stroke," *Revista de Neurologia*, vol. 34, no. 12, pp. 1127–1129, 2002.
[22] W. Küker, J. Weise, H. Krapf, F. Schmidt, S. Friese, and M. Bähr, "MRI characteristics of acute and subacute brainstem and thalamic infarctions: value of T2- and diffusion-weighted sequences," *Journal of Neurology*, vol. 249, no. 1, pp. 33–42, 2002.
[23] P. A. Ringleb, P. D. Schellinger, C. Schranz, and W. Hacke, "Thrombolytic therapy within 3 to 6 hours after onset of ischemic stroke: useful or harmful?" *Stroke*, vol. 33, no. 5, pp. 1437–1441, 2002.
[24] M. Uno, M. Harada, K. Yoneda, S. Matsubara, K. Satoh, and S. Nagahiro, "Can diffusion- and perfusion-weighted magnetic resonance imaging evaluate the efficacy of acute thrombolysis in patients with internal carotid artery or middle cerebral artery occlusion?" *Neurosurgery*, vol. 50, no. 1, pp. 28–35, 2002.
[25] H. J. Aronen, I. E. Gazit, D. N. Louis, et al., "Cerebral blood volume maps of gliomas: comparison with tumor grade and histologic findings," *Radiology*, vol. 191, no. 1, pp. 41–51, 1994.
[26] T. M. Ernst, L. Chang, M. D. Witt, et al., "Cerebral toxoplasmosis and lymphoma in AIDS: perfusion MR imaging experience in 13 patients," *Radiology*, vol. 208, no. 3, pp. 663–669, 1998.
[27] G. W. Albers, "Expanding the window for thrombolytic therapy in acute stroke: the potential role of acute MRI for patient selection," *Stroke*, vol. 30, no. 10, pp. 2230–2237, 1999.
[28] J. Röther, P. D. Schellinger, A. Gass, et al., "Effect of intravenous thrombolysis on MRI parameters and functional outcome in acute stroke < 6 hours," *Stroke*, vol. 33, no. 10, pp. 2438–2445, 2002.
[29] Q. Jiang, R. L. Zhang, Z. G. Zhang, et al., "Magnetic resonance imaging indexes of therapeutic efficacy of recombinant tissue plasminogen activator treatment of rat at 1 and 4 hours after embolic stroke," *Journal of Cerebral Blood Flow and Metabolism*, vol. 20, no. 1, pp. 21–27, 2000.
[30] H. Ay, J. Oliveira-Filho, F. S. Buonanno, et al., "'Footprints' of transient ischemic attacks: a diffusion-weighted MRI study," *Cerebrovascular Diseases*, vol. 14, no. 3-4, pp. 177–186, 2002.

**Ying Han** is an Associate Professor of neurology. She graduated in Harbin Medical University in 1988. From 1988 to 1994, she worked in the Department of Neurology, Shenyang Railway General Hospital, and from 1994 to 2005, worked in the Department of Neurology and Rehabilitation, Shougang Hospital, Beijing University. She has been studying in the Institute of Cognitive Neurosciences and Learning, Beijing Normal University since 2005.

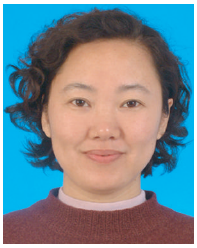

**Enzhong Li** is an Associate Professor of Radiology. He graduated in Harbin Medical University in 1982. From 1985 to 1994, he worked in the Department of Anatomy, Harbin Medical University. From 1994 to 2001, he worked in the Department of Magnetic Resonance Imaging, Shougang Hospital, Beijing University. From 2001 to 2003, he studied in the Institute of Automation, Chinese Academy of Sciences and then worked in the Associated Laboratory of Beijing Tiantan Hospital, Institute of Automation, and Chinese Academy of Sciences. Now he works in the National Natural Science Foundation of China.

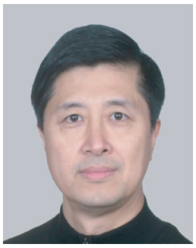

**Jie Tian** received the Ph.D. degree in artificial intelligence from the Institute of Automation, Chinese Academy of Sciences, in 1992. From 1994 to 1996, he was a postdoctoral fellow at the Medical Image Processing Group, University of Pennsylvania. Since 1997, he has been a Professor in the Institute of Automation, Chinese Academy of Sciences. His research interests are the medical image process and analysis, pattern recognition, bioinformatics, and so forth.

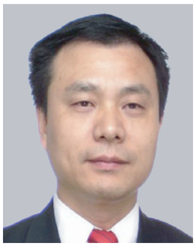

**Jian Chen** received the B.S. degree in electronic engineering from Beijing Normal University of China in 2003 and then went on a five-year doctoral program in medical image processing at Institute of Automation, Chinese Academy of Sciences.

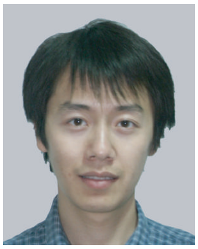

**Huifang Wang** received the Master's degree in clinical medicine from Hunan Medical University (in China) in 1995, received the Ph.D. degree in Peking University in 2005. Now she is an Associate Professor and works in the Department of Neurology, Shougang Hospital, Peking University. Her interests focus on neuroimaging and interventional therapy.

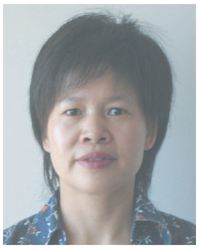

**Jianping Dai** is President of Beijing Tiantan Hospital, President of Chinese Radiological Society, Director Doctor, and a Ph.D. advisor. From 1979–1981, he went on a two-year studentship research program in neuroradiology and interventional radiology at Massachusetts General Hospital Harvard Medical School.

